# Stakeholder Perceptions of Animal Welfare as a Component of Sustainable Beef Programs in the United States—A Pilot Study

**DOI:** 10.3390/ani14091348

**Published:** 2024-04-30

**Authors:** Lily Edwards-Callaway, Melissa Davis, Lauren Dean, Brianna McBride

**Affiliations:** Department of Animal Sciences, Colorado State University, Fort Collins, CO 80523, USA; melissa.davis@colostate.edu (M.D.); lauren.dean@colostate.edu (L.D.); brianna.mcbride@colostate.edu (B.M.)

**Keywords:** agriculture, beef supply chain, cattle, sustainability, well-being

## Abstract

**Simple Summary:**

Cattle welfare is a critical component of sustainable beef production systems. The objective of this pilot study was to investigate how a specific group of beef industry stakeholders perceived and integrated animal welfare into sustainability programs using an online survey. Individuals represented owners, managers, and chief sustainability officers from a variety of stakeholder groups. Respondents recognized cattle welfare as a critical component to ensuring the overall sustainability of a production system and integrated animal welfare outcomes within their sustainability programs. It was evident that respondents felt animal care and providing basic needs to animals was a critical component of cattle welfare. The motivation of respondents to include cattle welfare in sustainability programming was related to cattle health and performance as well as consumer perception. Additionally, respondents often mentioned following best management practices and often referenced industry guidelines, assessment tools, and sometimes regulations where relevant. Respondents shared that the three pillars of sustainability (e.g., environmental, social, and economic) were interconnected, highlighting the need for balanced approaches to integrating sustainable animal welfare solutions in beef systems.

**Abstract:**

This study aimed to investigate how stakeholders in the United States beef industry incorporate animal welfare into their sustainability programs. A survey was administered online to the U.S. Roundtable for Sustainable Beef membership. Twenty-seven surveys were analyzed. Most respondents (*n* = 26, 96%) had sustainability programs that incorporated animal welfare. Most respondents believed that welfare positively impacted environmental (*n* = 25/26, 96%), economic (*n* = 25/26, 96%), and social (*n* = 26/26, 100%) sustainability. The thematic analysis of five free response questions identified ten themes: Animal Care, Regulations and Guidelines, Responsibility, Consumers and Stakeholders, Performance and Efficiency, Financial Impact, Connectedness, Critical Component, Animal-based Outcomes, and Employees. When asked to define welfare, the most common themes were Animal Care and Regulations and Guidelines. When asked why welfare was a component of their sustainability program, the top factors from a provided list were: cattle health (*n* = 20, 74%), cattle performance (*n* = 12, 44%), and consumer perceptions (*n* = 12, 44%). Findings suggest a widespread recognition of animal welfare’s importance within sustainable beef production.

## 1. Introduction

The cattle industry, on global, national, and local fronts, has engaged in exploring production practices and technologies to advance the sustainability of beef production systems [1,2,3]. The initiatives set forth by organizations such as the Global Roundtable for Sustainable Beef (GRSB) and the affiliated regional roundtables (e.g., U.S. Roundtable for Sustainable Beef; Brazilian Roundtable on Sustainable Livestock; Australian Beef Sustainability Framework) have elevated the attention to and awareness of the importance of the environmental, social, and economic components (i.e., the three pillars of sustainability) of beef production. Literature focusing on sustainability in animal agriculture has documented the importance of animal welfare and/or the need for the inclusion of welfare in sustainability programming [4,5,6,7,8,9]. Simultaneously, consumer interest in management practices that promote welfare of farm animals is continually growing [10,11,12]. There are many reasons why a production system may be deemed “unsustainable” (e.g., no market for the product, harmful environmental effects, pollution, and poor conditions for employees) and management practices that result in poor animal welfare conditions is among those reasons [13].

There is limited research exploring the intersection of the three pillars of sustainability, particularly with the objective of understanding how, or if, the emphasis of one pillar can deemphasize or even negatively impact another [14,15]. Place [14] discusses how a keen focus on the efficient use of resources in livestock production, i.e., the reduced use of natural resources coupled with increased animal protein output, could conflict with promoting animal welfare. Shields and Orme-Evans [15] suggested that animal welfare has not been adequately considered during the evaluation of certain management strategies that promote other aspects of sustainability, using climate change mitigation strategies as an example. There are trade-offs in agriculture systems that need to be considered to ensure a holistic approach to achieving sustainability goals. While Place [14] discusses the trade-offs made in systems, they also emphasize the importance of identifying synergies between components and identifying win–win scenarios in which an improvement in one pillar could benefit another. It would be valuable to understand how stakeholders perceive the interconnectedness between their environmental, social, and economic goals. Additionally, generally, animal welfare is included within the social pillar of sustainability but it would be beneficial to understand how stakeholders integrate welfare into their overall sustainability programs.

Promoting animal health and welfare is essential to sustainable livestock production systems [16,17]. Animal health and welfare are included as critical components in global sustainability platforms; both the global and regional roundtables for sustainable beef have designed priority indicator goals and sector targets for the advancement of sustainable beef production that include animal health and well-being metrics [18,19,20]. For example, one goal set by the GRSB is to increase animal care training opportunities by 25% from 2020 levels (i.e., levels are current benchmarking values of the member organizations) [21]. Additionally, the U.S. Roundtable for Sustainable Beef (USRSB) has set an overarching goal for the continuous improvement of animal health and well-being to be achieved through unique targets within cow-calf, auction market, feedyard, packer and processor, and retail and foodservice sectors [22]. Assessing animal welfare is complex and often requires a multifactorial approach to assessment. Currently, there exists little published data on a national scale characterizing how stakeholders are integrating and monitoring animal welfare outcomes within their sustainability programs.

To make improvements in cattle welfare within a sustainability framework, there needs to be grassroots adoption of and belief in animal welfare metrics. Currently, there is a lack of information outlining stakeholder perceptions about what animal welfare means to their operations within the context of sustainability. There is also an opportunity for more research and outreach to understand how the various cattle industry sectors integrate animal welfare into a holistic approach to sustainability programming. Additionally, as the beef supply chain continues to evaluate and implement management practices that have positive impacts for environmental and economic footprints, it is important to understand how stakeholders view the interrelationships between all pillars of sustainability. The objective of this research was to investigate how a specific group of United States beef industry stakeholders affiliated with the USRSB perceive and integrate animal welfare into sustainability programs.

## 2. Materials and Methods

This study was reviewed and deemed exempt by the Colorado State University (CSU) Institutional Review Board (#4702) prior to survey distribution.

### 2.1. Development of Survey and Study Population

This survey took place in September and October 2023, and was developed in an online survey software (Qualtrics, Provo, UT, USA). The survey was distributed by the USRSB using an electronic mailing list of 142 primary contacts of their member organizations. A direct link to the online survey was included in the recruitment email, and it was requested that only one person per organization or company responded to the survey. Two reminders to take the survey were sent out a week apart after the initial email request. On the day of the last reminder email, the survey link and description were also added to the USRSB newsletter that reached a wider audience. The survey was voluntary, and there was no incentive provided to participate. The only question respondents were forced to answer to continue was to obtain informed consent at the beginning of the survey.

The survey included 27 questions (Supplementary Material, Survey S1). The questions were focused on determining industry stakeholder incorporation and the perception of animal welfare within their sustainability programs, their methods and frequency of tracking animal welfare, and their perceptions about the relationships between animal welfare and the three pillars of sustainability. Question types consisted of dichotomous, multiple choice, multiple answer, free response, and Likert scale. Questions were asked at the beginning of the survey to establish the respondent’s role within the company or organization (i.e., owner, director, chief sustainability officer, manager, quality assurance specialist, or other), which constituency or stakeholder group they represented as categorized by USRSB (i.e., producers, auction market, allied industry, packers and processors, retail and food service, civil society, or other), and which category of annual gross revenue the company or organization falls under following the USRSB membership structure. The annual gross revenue is how USRSB categorizes the size of the member, so it was used for a similar purpose in this study. Few respondents answered this question so these results will not be reported.

### 2.2. Analysis

#### 2.2.1. Data Summarization

A total of 44 surveys were received. Seventeen were removed because they were less than 80% complete; many of these were less than one-third complete. The following analysis was performed on the final subset of 27 surveys. Answers to the any dichotomous, multiple-choice, multiple-answer, and Likert scale questions were summarized using descriptive statistics in Microsoft Excel (Microsoft Corporation, Redmond, WA, USA). There were a few questions that not all 27 respondents provided answers to; these are noted by a different total when being reported.

#### 2.2.2. Qualitative Analysis

Thematic analysis was conducted on five of the open-response survey questions. The questions were as follows: (Q1) “How do you as a representative of your company or organization define animal welfare within the context of beef production?”; (Q2) “Why is animal welfare a component of your sustainability program?”; (Q3) “Considering animal welfare is a component of your sustainability program, what types of indicators do you use to measure or track animal welfare within that program?”; (Q4) “Why do you believe that emphasizing one pillar of sustainability (environment, economic, or social) negatively impacts either of the other two?”; (Q5) “Why do you not believe that emphasizing one pillar of sustainability (environment, economic, or social) negatively impacts either of the other two?” Analysis was performed according to the methods described by Braun and Clarke [23]. 

All co-authors reviewed the survey responses individually and created short descriptors to describe the responses. Co-authors met and refined and combined the descriptors into broader themes to develop a codebook. Using the codebook, three researchers independently coded each of the responses (e.g., assigned a theme). These three coders had varying levels of experience in animal welfare. One coder is a faculty member at the university conducting research in animal welfare and has past experience working in different sectors of the beef industry. The second coder has her doctoral degree in Animal Science, specializing in animal welfare and meat science and continues to participate in a variety of welfare-focused research. The third coder has her bachelor’s degree in Dairy Science, has worked previously in the dairy industry, and is now a research associate at the university.

The full agreement of all coders was needed on the responses to each question before moving on to the next to validate the coding. Typically, two of the three coders were in full agreement for each response while the third coder was in partial agreement (i.e., the coder had either one less or one additional theme present on a multi-themed response); any differences in identified themes were discussed, and full agreement was reached on every response for all five survey questions. 

## 3. Results

The initial distribution of the survey to a listserv of 142 primary contacts for USRSB members resulted in 18 responses, namely a 12.7% response rate. The remaining 9 responses, for an overall total of 27, were collected after the survey was distributed to both the primary contacts in a reminder and within a newsletter sent to a wider audience, therefore, the authors cannot calculate a more specific response rate for this survey. 

The characteristics of the sample population are shown in Table 1. Respondents represented all constituency classifications with producers representing the largest group of respondents (*n* = 13, 48%). Within the producer group, two respondents (15%) were solely affiliated with the feedyard sector, six (46%) were affiliated with solely the cow-calf sector and the remaining respondents were involved with multiple segments of production (*n* = 5, 38%, e.g., cow/calf, feedyard, and/or stocker/backgrounder). Roles of the respondents varied, and a few individuals (*n* = 3) held multiple roles within their companies or operations. The most common roles represented in the sample population were owners (*n* = 8, 30%), followed by directors (*n* = 7, 26%) and managers (*n* = 7, 26%). 

The majority of respondents “agreed” or “strongly agreed” that “animal welfare is an important component of a sustainable beef production system” (*n* = 7, 26% and *n* = 17, 63%, respectively). Three (11%) respondents selected “strongly disagree” in response to this statement. The majority of respondents (*n* = 21, 78%) indicated that they “have a sustainability program through which they monitor, verify, or track improvement in sustainability metrics specifically related to their (company’s) role in beef production”. All but one respondent (*n* = 26, 96%) indicated that animal welfare was a component of their sustainability program. When asked to select factors that influenced their decision to incorporate animal welfare into a sustainability program from a provided list, cattle health (*n* = 20, 74%), cattle performance (*n* = 12, 44%), and consumer perceptions (*n* = 12, 44%) were the most selected factors (Table 2). When asked how often they reported animal welfare indicators per year, approximately half the respondents (*n* = 10 of 19, 53%) indicated that metrics were reported once per year. 

Over half of the respondents (*n* = 16, 59%) believed that emphasizing one pillar of sustainability negatively impacts either of the other two. One respondent selected “I don’t know” and ten respondents (37%) indicated that they did not believe emphasizing one pillar would impact the others. Follow-up free-response questions (Q4 and Q5) were asked after this question and are described in the qualitative analysis section of these results. Almost all individuals indicated that animal welfare positively impacts environmental (*n* = 25, 96%), economic (*n* = 25, 96%), and social (*n* = 26, 100%) sustainability; one respondent did not answer these questions. 

### Qualitative Analysis 

There were 10 themes identified through thematic analysis. Their definitions and sample survey responses are shown in Table 3. The results of this analysis are reported by question, below.

Q1: “How do you as a representative of your company or organization define animal welfare within the context of beef production?”

The most common theme found in the responses to this question was Animal Care; within this theme, Management and Animal Needs were the most frequently mentioned and were often both found within the same response. Many of the responses coded as Management included phrases related to “*following best management practices and stewardship*” and “*taking proper care of all animals*”. Animal Needs focused on specific aspects necessary to ensure animal welfare such as providing water and feed and an appropriate environment. Regulations and Guidelines was another common theme found in responses to this question often with reference to specific guidelines or organizations; Beef Quality Assurance (BQA) was the most common program mentioned.

Q2: “Why is animal welfare a component of your sustainability program?”

All but two themes, Animal-based Outcomes and Employees, were present in the responses to this question. The subtheme of Management, Responsibility, and Consumers and Stakeholders themes were the most common, often present together in some combination of the three within one response. The responses coded as Management often referenced animal welfare being part of their overall management program (e.g., “*it is an important component when implementing a holistic approach as far as your overall management program*” and “*to provide care for all animals under our stewardship*”). Many responses coded as Responsibility referenced the “*ethical responsibility*” to provide the best care to the animals. The responses coded as Consumers and Stakeholders referenced how animal welfare as part of a sustainability program is what consumers or other stakeholders are expecting and demanding (e.g., “*it is how most consumers view sustainably raised products*” and “*meet and exceed consumer expectations and demands*”).

Q3: “Considering animal welfare is a component of your sustainability program, what types of indicators do you use to measure or track animal welfare within that program?”

The themes of Animal Care and Regulations and Guidelines were the most frequently mentioned in response to this question and in general, more than one theme was present within each response. Over one-third of the responses had three or more themes present. Within Animal Care, the subtheme of Health and Veterinary Care was mentioned the most and included phrases such as “*work with our veterinarian on herd health*”, “*vaccines and illnesses*”, and “*animal health*”. Responses within the Regulations and Guidelines theme generally referred to requiring certifications, such as BQA, and adhering to guidelines, such as the North American Meat Institute. Additionally, third-party and/or internal audits were mentioned by multiple respondents as a mechanism for tracking animal welfare indicators.

Q4 and Q5: These questions were follow-up questions to the question “Do you believe that emphasizing one pillar of sustainability (environment, economic, or social) negatively impacts either of the other two?”; if respondents answered “yes”, they were directed to Q4, and if respondents answered “no”, they were directed to Q5. Sixteen respondents answered Q4 and ten respondents answered Q5.
Q4: “Why do you believe that emphasizing one pillar of sustainability (environment, economic, or social) negatively impacts either of the other two?”

Although several themes were present in the responses to this question, the majority of responses were coded as Connectedness. Almost all the respondents referenced how the three pillars of sustainability are interrelated and shared comments such as: “*sustainability is an optimization problem, and an optimal solution generally requires gives and takes*” and “*because in order for your overall program to be successful you must have all three working together. When we over emphasis’ one aspect over others it leads to undesirable outcomes*”.

Q5: “Why do you not believe that emphasizing one pillar of sustainability (environment, economic, or social) negatively impacts either of the other two?”

Similarly, the majority of responses to this question were coded as Connectedness with examples including statements such as: “*I think these pillars can work in harmony for the benefit of all” and “they are synergistic rather than cannibalistic*”. Additionally, there were several instances of the theme Critical Component. These comments highlighted the importance of all pillars of sustainability (e.g., “*all are equally important*”) and were always found with the Connectedness themes.

## 4. Discussion

The group surveyed in this study was USRSB membership, an organization whose mission is to “advance, support and communicate continuous improvement of sustainability across the U.S. beef value chain” [19]. The results summarized in this paper are representative of a small sample of individuals and their respective companies who are active in the sustainability space. Although the opinions shared in this paper may not be representative of the general population of beef industry stakeholders in the United States, the findings from this survey are valuable as they provide a preliminary view of how welfare is being integrated into sustainability programs within a progressive subgroup of the beef industry. Creative ways to incentivize survey participation should be explored in future research. Although online surveys are a cost-effective and an easy way to distribute surveys to a large audience [24], surveys have the potential to introduce bias (e.g., recall, self-reporting, social desirability) [25,26]. Additionally, attrition (i.e., not completing the survey), which occurred in this survey, is another challenge with online surveys [26].

Respondents represented all USRSB constituency classifications with Producers representing the largest group of respondents (*n* = 13, 48%). This aligns with the USRSB membership distribution; the largest membership segment is producers [27]. The number of cattle producers in the United States outweighs the number of allied industry members and packers and processors, and therefore, it is expected that the producer group was more heavily represented. Additionally, producers are directly linked to on-farm animal management and therefore likely have a strong appreciation and understanding of the role that animal welfare has in sustainable beef production systems. The most common roles were Owners, Directors, or Managers which is line with the characteristics of who received the survey, i.e., primary contacts for a member company of the USRSB. Additional demographics of survey respondents were not collected but the inclusion of other characteristics, such as gender, age, and education, are warranted in future work to understand how these factors may impact the perceptions of animal welfare.

It is evident from the results that respondents agreed that animal welfare was an important component of sustainable beef production systems. When asked how respondents defined animal welfare, the most common responses were related to Animal Care, specifically the subthemes of Management and Animal Needs. These responses are in line with generally accepted definitions of animal welfare (e.g., WOAH, [28]) and frameworks used to evaluate animal welfare (e.g., Five Freedoms, [29]; Five Domains, [30]). Considering that the majority of respondents were producers, these responses also align with daily responsibilities of animal owners and caretakers, i.e., caring for the animals, practicing low-stress procedures, and providing animals with needs such as food, water, preventative health care, and shelter. Regulations and Guidelines was another common theme found in responses to this question often with reference to specific guidelines or organizations. Many respondents referenced specific industry guidelines relevant to their industry sector indicating that they followed those guidelines or required certification in those programs. For example, the National Cattlemen’s Beef Association (NCBA) Beef Quality Assurance (BQA) Program [31] was mentioned frequently; respondents mentioned using BQA certifications and/or adhering to BQA guidelines to ensure animal welfare within their systems. The BQA program is a nationally recognized voluntary education program that provides information on good husbandry practices to producers for cattle care across different industry sectors (e.g., cow-calf, stocker, feedlot). The program provides mechanisms to become BQA certified and audit tools to conduct an internal assessment and external audits. The USRSB has established sector metrics and targets for animal health and well-being [22] and for all live animal sectors, the goal is to increase the number of individuals trained and certified in BQA or an equivalent program. Considering that the survey population comprised USRSB members, it is understandable that the BQA guidelines would be commonly referenced in these responses.

Although almost all the respondents indicated that animal welfare was a component of their sustainability program, approximately 20% of respondents said they did not monitor, verify, or track improvement in sustainability metrics. It is possible that these respondents may have sustainability programs in their infancy and are not yet measuring, monitoring, or tracking animal welfare metrics in a formal manner. Approximately half of the respondents indicated that welfare metrics were reported once per year. There is limited literature outlining the appropriate frequency for monitoring animal welfare outcomes in beef production systems; there is some literature that focuses on credible animal welfare monitoring schemes rather than frequency of measurement specifically [32]. Generally, corporations report aggregate data on an annual basis, for example in Sustainability Reports or Corporate Social Responsibility Reports. Many of the animal care guidelines recommend performing frequent self-assessments and audits (i.e., weekly or quarterly) and likely results from these more frequent assessments are summarized for annual reports. Additionally, many certified programs perform audits of enrolled livestock production facilities on an annual basis as part of verifying program adherence (e.g., Certified Animal Welfare Approved, [33]; GAP, [34]).

Consumer perception was one of the common factors selected when respondents indicated what factors influenced their decision to incorporate animal welfare into a sustainability program from a provided list. Similarly, Consumers and Stakeholders constituted one of the most common themes present in response to the free-response question “Why is animal welfare a component of your sustainability program?”. With the increased interest in where food comes from, the concern for and value of farm animal welfare among consumers continues to be prominent across society on a global scale [10,11,12,35,36]. Although many factors (e.g., gender, age, religion, geographic location, species) can impact perceptions of farm animal welfare, generally consumers expect and want high standards of animal care for animals within the food supply chain [12,37,38]. Broom [5] considers animal welfare in the context of sustainability and suggests that the animal welfare status of an animal production system informs personal decisions as to whether that system is sustainable (i.e., a system with good welfare is sustainable and vice versa). Both sustainability and animal welfare are considered credence attributes which are intangible food attributes that consumers cannot directly evaluate at the time of purchase without additional information [39]; credence attributes are different from search (e.g., price, color) and experience (e.g., taste) attributes which can be easily assessed before and after consumption of a product [40]. Credence attributes have been shown to influence purchasing decisions and willingness to pay for a variety of food products (e.g., organic foods, [41]; sustainability labelled chocolate, [42]; fair trade, [43]; sustainable foods, [44] including meat products (beef from climate friendly production practices, [45]; welfare friendly meat, [46]).

Cattle Health and Cattle Performance were also factors identified herein that influenced the decision to incorporate animal welfare into sustainability programs. The physical health (i.e., biological functioning) of an animal has always been one of the core components of animal welfare assessment [47,48]. In production systems, it is essential that cattle are healthy and productive (e.g., growing, maintaining pregnancies, free of disease) and thus it is not surprising that these two factors were identified as important to sustainability programs. Additionally, efficiency is often included as a critical criterion of sustainable beef systems [18]; presumably, ensuring cattle health can be one way to maximize production efficiency. By identifying Cattle Health and Performance as key reasons to incorporate welfare into sustainability programs, it demonstrates the beef industry’s acceptance and acknowledgment that welfare is critical to ensuring productive animal systems. The theme of Health and Veterinary Care was also frequently included in responses to the free response question about what types of indicators are included in respondents’ sustainability programs.

“Regulations” was not provided as an option when asking respondents about factors influencing the incorporation of animal welfare into sustainability programs, and in retrospect, perhaps it should have been. In the United States, there are no federal regulations that exist dictating on-farm animal care practices, and therefore, regulations may not have been identified as highly influential. If this had been a global survey, it certainly would be relevant to include regulations and laws as a driving factor as animal care laws exist in other countries. Two federal regulations in the United States exist that focus on welfare during transportation (e.g., The Twenty Eight Hour Law, [49]) and humane handling and slaughter (The Humane Methods of Slaughter Act, [50]) and slaughter plant regulations were specifically mentioned in some individual responses as reasons that animal welfare was included in their sustainability programs. A quarter of respondents did select Risk Avoidance as an influential factor for welfare inclusion in programming, and although not directly asked, compliance with regulations could be considered a critical component of avoiding risk.

Almost all individuals indicated that animal welfare positively impacts environmental, economic, and social sustainability. Over half of the respondents believed that emphasizing one pillar of sustainability negatively impacts either of the other two. Follow-up free-response questions were included after this question asking respondents to explain why or why not. In response to these questions, respondents identified how there are trade-offs between the pillars of sustainability, how they need to be considered together, and how emphasizing one aspect over another could lead to unfavorable sustainability outcomes. There are several examples demonstrating this interconnectedness of environmental, social, and economic components of sustainable beef systems often in the context of evaluating trade-offs and synergies. Consider the relationship between animal welfare and environmental sustainability for example, a reduction in environmental impact by intensifying production systems could potentially lead to restrictions on animal movement, inhibit natural behaviors, and reduce social contact. Trade-off analysis is not a new concept in agriculture [51,52], but perhaps could be used more intentionally to inform sustainability initiative decision-making at the facility level. Conversely, there are also synergies between the pillars of sustainability in which improvement in one area could similarly provide benefits in another. For example, reducing the prevalence of bruising in cattle would be a welfare benefit and improve the economic viability of a system due to reduced economic loss from reduced yield. Perhaps capitalizing on some of the positive impacts improving cattle welfare could have on environmental and economic outcomes of an operation, i.e., identifying the “win–win” solutions and synergies [14], is a valuable approach to evaluating sustainability initiatives moving into the future.

## 5. Conclusions

This survey provides valuable preliminary information about stakeholders’ perceptions of animal welfare within the context of sustainable beef systems. It is evident that this group of stakeholders from across the beef supply chain agrees that welfare is an integral component to beef sustainability. Respondents identified welfare as being multi-faceted and encompassing many factors related to animal needs. Throughout the survey responses to many of the questions, the important role of industry guidelines for the implementation of best management practices was clearly emphasized. It is critical that the holistic evaluation of trade-offs between the pillars of sustainability is performed considering the implementation of new management practices. Future research should focus on exploring the perceptions of welfare within sustainability on a global scale. Additionally, future efforts should focus on understanding the synergies that exist between animal welfare improvements and environmental and economic outcomes; identifying win–win opportunities will help the beef industry identify effective solutions to improve sustainability within beef systems.

## Figures and Tables

**Table 1:** Respondent characteristics (*n* = 27).

Constituency ^1^	*n*	%
Allied Industry	6	22%
Civil Society	1	4%
Packers and Processors	4	15%
Producers	13	48%
Retail & Food Service	2	7%
Other	2	8%
**Role ^2^**		
Chief Sustainability Officer	5	19%
Director	7	26%
Manager	7	26%
Owner	8	30%
Other	3	11%

^1^ One respondent selected two constituencies; ^2^ Three individuals selected multiple roles.

**Table 2:** Respondent selections to the following question: Which of the following factors would or did influence the decision to incorporate animal welfare into a sustainability program? Please select your top three.

Factors	*n*	%
Cattle Health	20	74%
Cattle Performance	12	44%
Consumer Perceptions	12	44%
Environmental Impact	6	22%
Human Health	2	7%
Marketing Differentiation/Advantage	3	11%
Production Efficiency	9	33%
Product Quality	7	26%
Risk Avoidance	6	22%
Technology and Innovation	2	7%
Worker Satisfaction	1	4%
Other	1	4%

**Table 3:** Themes, definitions, and examples of responses for all themes identified within the thematic analysis. The questions ^1^ that each theme was found in is also noted.

Theme (Subtheme)	Definition	Questions That These Themes Were Present in the Responses	Primary Examples from the Responses That Fit Each Theme
Animal Care	Comments related to many different aspects of animal care and needs, defined by subthemes below.		
*Management*	Comments related to general best management procedures for caring for animals (e.g., stewardship, stockmanship, basic animal care). Mention of procedures that directly impact animal welfare (e.g., castration, physical alterations).	Q1, Q2, Q3	*“We define animal welfare as cattle care, and we look to best management practices and stewardship as a way to ensure positive animal welfare.”* *“Taking proper care of all animals that enter our plant.”* *“Animal welfare incorporates…physical alterations…”*
*Animal Needs*	Comments regarding basic needs of animals (e.g., water, shelter, environment, five freedoms, comfort).	Q1, Q2, Q3	*“The ability for animals to express the five freedoms.”* *“Making sure all livestock have abundant sources of water and feedstuffs…”* *“…pen conditions…”*
*Health and Veterinary Care*	References to animal health (e.g., morbidity, mortality, death loss, illness) and veterinary care (e.g., vaccines, preventative medicine).	Q1, Q2, Q3, Q4	*“Improving the health…of livestock.”* *“Veterinary satisfaction, efficacy and safety studies and product valuation on health.”* *“Treatment success rate, overall treatment rate, death loss.”*
Animal Handling	Mentions of animal handling techniques (e.g., low stress, humane)	Q1, Q2, Q3	*“Handling of animals during transportation and slaughter”* *“Low stress…”* *“…animal handling observations (falls, prod use, mis-catch, vocalizations, etc.)…”*
Regulations and Guidelines	Mentions of any certification, auditing, regulations, guidelines, or organizations related to animal care/welfare (e.g., BQA/BQAT, NAMI, third-party, USDA, USRSB).	Q1, Q2, Q3	*“We use the USRSB definition.”* *“We use BQA certifications and/or other accredited animal welfare programs or third-party audits as welfare indicators.”* *“We adhere to NAMI Guidelines and metrics to Humane Handling of Livestock.”*
Responsibility	Comments related to the moral and ethical responsibility of cattle industry stakeholders to upholding animal welfare (e.g., duty, what is right).	Q1, Q2	*“Cattle producers have the responsibility…”* *“A moral and ethical responsibility.”* *“It’s about making the right choices…”*
Consumers and Stakeholders	References to the value that members of the community place on animal care/welfare.	Q2	*“…our consumers are extremely passionate about it…”* *“Consumer data shows that animal welfare is a key topic and concern…”* *“AW [animal welfare] is a top priority of our stakeholders, internal and external.”*
Performance and Efficiency	Statements related to impacts on animal performance (e.g., growth, product quality) and efficiency.	Q1, Q2, Q3, Q5	*“…responsible animal care contributes to resource efficiency…”* *“Sustainability is multifactorial—animal welfare, production efficiency…”* *“To perform up to their potential…”*
Financial Impact	Anything that economically impacts the company/organization.	Q1, Q2, Q4, Q5	*“Basic animal welfare can’t be decoupled from profitability.”* *“Working on environmental issues on the ranch benefits our economic positions and via solid financial conditions we are able to contribute significantly to our communities.”*
Connectedness	Statements related to the balance, interconnectedness, and synergy between pillars of sustainability; mention of trade-offs, equality, together, harmony, crossover, or holistic approach.	Q2, Q4, Q5	*“…all interwoven into what makes us sustainable.”* *“…an important component when implementing a holistic approach…”* *“…making well balanced decisions.”*
Critical Component	Animal welfare is a critical component or the foundation of sustainability. Any pillar of sustainability is labeled as important, critical, or foundational.	Q2, Q4, Q5	*“Because it’s a critical component of sustainability.”* *“…animal welfare is not just a component, it is the foundation.”* *“…they are all three connected and equally as important.”*
Animal-based Outcomes	Mentions of using animal-based outcomes as indicators of animal welfare (e.g., BCS, behavior, mobility, ADG).	Q1, Q3	*“…behavior of animals under people’s control…”* *“…animal based outcomes (BCS, lameness, injuries)…”*
Employees	References to company employees or animal caretakers including training, safety, and attitude.	Q1, Q3, Q4	*“BQA certification for all who handle livestock.”* *“Caretaker training/BQA and BQAT certification…”* *“Attitude and actions of employees.”*

^1^ Q1: How do you as a representative of your company or organization define animal welfare within the context of beef production?; Q2: Why is animal welfare a component of your sustainability program?; Q3: Considering animal welfare is a component of your sustainability program, what types of indicators do you use to measure or track animal welfare within that program?; Q4: Why do you believe that emphasizing one pillar of sustainability (environment, economic, or social) negatively impacts either of the other two?; Q5: Why do you not believe that emphasizing one pillar of sustainability (environment, economic, or social) negatively impacts either of the other two?

## Data Availability

Data are available upon request to the authors.

## Supplementary Materials

The following supporting information can be downloaded at: https://www.mdpi.com/article/10.3390/ani14091348/s1, Survey S1: Survey tool.

## References

[1] Maia de Souza D., Petre R., Jackson F., Hadarits M., Pogue S., Carlyle C.N., Bork E., McAllister T. (2017). A review of sustainability enhancements in the beef value chain: State-of-the-art and recommendations for future improvements. Animals.

[2] Pulina G., Acciaro M., Atzori A.S., Battacone G., Crovetto G.M., Mele M., Pirlo G., Rassu S.P.G. (2021). Animal board invited review–Beef for future: Technologies for a sustainable and profitable beef industry. Animal.

[3] Greenwood P.L. (2021). An overview of beef production from pasture and feedlot globally, as demand for beef and the need for sustainable practices increase. Animal.

[4] Phillips C.J.C., Sorensen J.T. (1993). Sustainability in Cattle Production Systems. J. Agric. Environ. Ethics.

[5] Broom D.M. (2010). Animal Welfare: An Aspect of Care, Sustainability, and Food Quality Required by the Public. J. Vet. Med. Educ..

[6] Garnett T., Appleby M.C., Balmford A., Bateman I.J., Benton T.G., Bloomer P., Burlingame B., Dawkins M., Dolan L., Fraser D. (2013). Sustainable Intensification in Agriculture: Premises and Policies. Science.

[7] Tucker C.B., Mench J.A., von Keyserlingk M.A.G., Kebreab E. (2013). Animal Welfare: An Integral Component of Sustainability. Sustainable Animal Agriculture.

[8] Peralta J.M., Reynolds J., Kerr C.V., Thompson P.B., Kaplan D.M. (2019). Sustainability and Animal Agriculture. Encyclopedia of Food and Agricultural Ethics.

[9] Schneider F., Tarawali S. (2021). Sustainable Development Goals and Livestock Systems. Rev. Sci. Technol. OIE.

[10] European-Commission (2016). Attitudes of EU Citizens towards Animal Welfare, Report.

[11] Alonso M.E., González-Montaña J.R., Lomillos J.M. (2020). Consumers’ concerns and perceptions of farm animal welfare. Animals.

[12] McKendree M.G., Croney C.C., Widmar N.O. (2014). Effects of demographic factors and information sources on United States consumer perceptions of animal welfare. J. Anim. Sci..

[13] Broom D.M. (2017). Components of sustainable animal production and the use of silvopastoral systems. Rev. Bras. Zootec..

[14] Place S.E., Mench J. (2018). Animal welfare and environmental issues. Advances in Agricultural Animal Welfare.

[15] Shields S., Orme-Evans G. (2015). The impacts of climate change mitigation strategies on animal welfare. Animals.

[16] Broom D.M. (2021). A method for assessing sustainability, with beef production as an example. Biol. Rev..

[17] Broom D.M. (2019). Animal welfare complementing or conflicting with other sustainability issues. Appl. Anim. Behav. Sci..

[18] Global Roundtable for Sustainable Beef. https://grsbeef.org/.

[19] U.S. Roundtable for Sustainable Beef. https://www.usrsb.org/.

[20] European Roundtable for Beef Sustainability. https://saiplatform.org/erbs/.

[21] Global Beef Sustainability Goal: Animal Health and Welfare. https://grsbeef.org/sustainability-goals/animal-health-welfare/.

[22] USRSB High-Priority Indicator Goals & Sector Targets. https://www.usrsb.org/goals#animalHealthAndWellBeing.

[23] Braun V., Clarke V. (2006). Using thematic analysis in psychology. Qual. Res. Psychol..

[24] Fenner K., Hyde M., Crean A., McGreevy P. (2020). Identifying sources of potential bias when using online survey data to explore horse training, management, and behaviour: A systematic literature review. Vet. Sci..

[25] Althubaiti A. (2016). Information bias in health research: Definition, pitfalls, and adjustment methods. J. Multidiscip. Healthc..

[26] Ward M.K., Meade A.W., Allred C.M., Pappalardo G., Stoughton J.W. (2017). Careless response and attrition as sources of bias in online survey assessments of personality traits and performance. Comput. Hum. Behav. Rep..

[27] U.S. Roundtable for Sustainable Beef. 2022 Annual Report. https://beef.widen.net/content/bcokxif4vj/original/2022-USRSB-AnnualReport-final.pdf?u=q5atpk&download=true.

[28] Terrestrial Animal Health Code. World Organisation for Animal Health. 2023, Section 7, Chapter 7.1. https://www.woah.org/en/what-we-do/standards/codes-and-manuals/terrestrial-code-online-access/?id=169&L=1&htmfile=titre_1.7.htm.

[29] Farm Animal Welfare Council (FAWC) (1993). Second Report on Priorities for Research and Development in Farm Animal Welfare.

[30] Mellor D.J., Reid C.S.W., Baker R.M., Jenkin G., Mellor D.J. (1994). Concepts of animal well-being and predicting the impact of procedures on experimental animals. Improving the Well-Being of Animals in the Research Environment.

[31] Beef Quality Assurance National Manual National Cattlemen’s Beef Association. https://www.bqa.org/Media/BQA/Docs/bqa_manual_final.pdf.

[32] Barnett J.L., Hemsworth P.H. (2009). Welfare monitoring schemes: Using research to safeguard welfare of animals on the farm. J. Appl. Anim. Welf. Sci..

[33] Certified Animal Welfare Approved by AGW. https://agreenerworld.org/certifications/animal-welfare-approved/.

[34] G.A.P. Certification. G.A.P. Certification—Global Animal Partnership. https://globalanimalpartnership.org/certification.

[35] Kendall H.A., Lobao L.M., Sharp J.S. (2006). Public concern with animal well-being: Place, social structural location, and individual experience. Rural. Sociol..

[36] Sweeney S., Regan Á., McKernan C., Benson T., Hanlon A., Dean M. (2022). Current consumer perceptions of animal welfare across different farming sectors on the island of Ireland. Animals.

[37] Prickett R., Norwood F.B., Lusk J. (2010). Consumer Preferences for Farm Animal Welfare: Results from a Telephone Survey of US Households. Anim. Welf..

[38] Sinclair M., Lee N.Y.P., Hötzel M.J., De Luna M.C.T., Sharma A., Idris M., Derkley T., Li C., Islam M.A., Iyasere O.S. (2022). International perceptions of animals and the importance of their welfare. Front. Anim. Sci..

[39] Darby M.R., Karni E. (1973). Free competition and the optimal amount of fraud. J. Law Econ..

[40] Janssen M., Hamm U. (2012). Product labelling in the market for organic food: Consumer preferences and willingness-to-pay for different organic certification logos. Food Qual. Prefer..

[41] Katt F., Meixner O. (2020). A systematic review of drivers influencing consumer willingness to pay for organic food. Trends Food Sci. Technol..

[42] Vecchio R., Annunziata A. (2015). Willingness-to-pay for sustainability-labelled chocolate: An experimental auction approach. J. Clean. Prod..

[43] Konuk F.A. (2019). Consumers’ willingness to buy and willingness to pay for fair trade food: The influence of consciousness for fair consumption, environmental concern, trust and innovativeness. Food Res. Int..

[44] Kovacs I., Keresztes E.R. (2022). Perceived consumer effectiveness and willingness to pay for credence product attributes of sustainable foods. Sustainability.

[45] Li X., Jensen K.L., Clark C.D., Lambert D.M. (2016). Consumer willingness to pay for beef grown using climate friendly production practices. Food Policy.

[46] Miranda-de La Lama G.C., Estévez-Moreno L.X., Sepúlveda W.S., Estrada-Chavero M.C., Rayas-Amor A.A., Villarroel M., María G.A. (2017). Mexican consumers’ perceptions and attitudes towards farm animal welfare and willingness to pay for welfare friendly meat products. Meat Sci..

[47] Fraser D., Weary D.M., Pajor E.A., Milligan B.N. (1997). A scientific conception of animal welfare that reflects ethical concerns. Anim. Welf..

[48] Broom D.M. (1991). Animal welfare: Concepts and measurement. J. Anim. Sci..

[49] (1994). Twenty-Eight Hour Law. United States Code: Transportation of Animals. 49 U.S. Code § 80502—Transportation of Animals. LII/Legal Information Institute. https://www.law.cornell.edu/uscode/text/49/80502.

[50] (1979). Code of Federal Regulations. Humane Slaughter of Livestock. https://www.law.cornell.edu/cfr/text/9/part-313.

[51] Kanter D.R., Musumba M., Wood S.L.R., Palm C., Antle J., Balvanera P., Dale V.H., Havlik P., Kline K.L., Scholes R.J. (2018). Evaluating agricultural trade-offs in the age of sustainable development. Agric. Syst..

[52] Klapwijk C., Van Wijk M., Rosenstock T., Van Asten P., Thornton P., Giller K. (2014). Analysis of trade-offs in agricultural systems: Current status and way forward. Curr. Opin. Environ. Sustain..

