# Supplementary material for: Stakeholder Perceptions of Animal Welfare as a Component of Sustainable Beef Programs in the United States—A Pilot Study
Source: Animals (Basel). 2024 Apr 30;14(9):1348. doi: 10.3390/ani14091348 (PMC11083033; doi:10.3390/ani14091348)
Supplement: Supplementary file 1 [file animals-14-01348-s001.zip › animals-2975650-supplementary.pdf]

Animal Welfare & Sustainability Survey

Q1 To which of the following roles do you identify with within your company or organization? Please select all that apply.

- ☐ Owner (1)
- ☐ Director (2)
- ☐ Chief Sustainability Officer (3)
- ☐ Manager (4)
- ☐ Quality Assurance Specialist (5)
- ☐ Other (6) ____

Q2 Which of the following constituencies (i.e., stakeholder group) do you represent? Please select all that apply.

- ☐ Producers (1)
- ☐ Auction Market (7)
- ☐ Allied Industry (2)
- ☐ Packers & Processors (3)
- ☐ Retail & Food Service (4)
- ☐ Civil Society (5)
- ☐ Other (6) ____

Q3 Which of the following producer segments would you consider yourself? Please select all that apply.

- ☐ Cow/Calf (1)
- ☐ Stocker/Backgrounder (2)
- ☐ Feedyard (3)

Q4 Following the USRSB membership structure, which category of annual gross revenue do you fall under within the producer constituency?

- ○ Individual (1)
- ○ Associations < $2 million (2)
- ○ Associations $2 million - $10 million (3)
- ○ Associations > $10 million (4)

Q5 Following the USRSB membership structure, which category of annual gross revenue do you fall under within the auction market constituency?

- ○ Individual (1)
- ○ Associations < $2 million (2)
- ○ Associations $2 million - $10 million (3)
- ○ Associations > $10 million (4)

Q6 Following the USRSB membership structure, which category of annual gross revenue do you fall under within the allied industry constituency?

- < $5 million (1)
- $5 million - $100 million (2)
- > $100 million (3)
- Associations < $2 million (4)
- Associations $2 million - $10 million (5)
- Associations > $10 million (6)

Q7 Following the USRSB membership structure, which category of annual gross revenue do you fall under within the packers and processors constituency?

- < $5 million (1)
- $5 million - $100 million (2)
- > $100 million (3)
- Associations < $2 million (4)
- Associations $2 million - $10 million (5)
- Associations > $10 million (6)

Q8 Following the USRSB membership structure, which category of annual gross revenue do you fall under within the retail and food service constituency?

- ○ < $5 million (1)
- ○ $5 million - $100 million (2)
- ○ > $100 million (3)
- ○ Associations < $2 million (4)
- ○ Associations $2 million - $10 million (5)
- ○ Associations > $10 million (6)

Q9 Following the USRSB membership structure, which category of annual gross revenue do you fall under within the civil society constituency?

- ○ < $5 million (1)
- ○ $5 million - $100 million (2)
- ○ > $100 million (3)

Q10 What is your approximate annual gross revenue within the "other" constituency?

____

Q11 Do you have a sustainability program through which you monitor, verify, or track improvement in sustainability metrics specifically related to your (company's) role in beef production?

- ○ Yes (1)
- ○ No (2)
- ○ I do not know (3)

Q12 Is animal welfare a component of your sustainability program?

- ◯ Yes (1)
- ◯ No (2)
- ◯ I do not know (3)

Q13 How do you as a representative of your company or organization define animal welfare within the context of beef production?

____

Q14 To what level do you agree/disagree with the following statement: Animal welfare is an important component of a sustainable beef production system.

- ◯ Strongly Disagree (1)
- ◯ Disagree (2)
- ◯ Agree (3)
- ◯ Strongly Agree (4)

Q15 Why is animal welfare a component of your sustainability program?

____

Q16 Why is animal welfare not a component of your sustainability program?

____

Q17 Considering animal welfare is a component of your sustainability program, what types of indicators do you use to measure or track animal welfare within that program? Please type N/A if not applicable.

____

Q18 Considering animal welfare is a component of your sustainability program, how often are your animal welfare indicators reported per year? Please type N/A if not applicable.

____

Q19 Which of the following factors would or did influence the decision to incorporate animal welfare into a sustainability program? Please select your top three.

- ▢ Cattle Health (1)
- ▢ Cattle Performance (3)
- ▢ Consumer Perceptions (4)
- ▢ Environmental Impact (10)
- ▢ Human Health (2)
- ▢ Marketing Differentiation/Advantage (8)
- ▢ Production Efficiency (6)
- ▢ Product Quality (5)
- ▢ Risk Avoidance (9)
- ▢ Technology and innovation (7)
- ▢ Worker Satisfaction (11)
- ▢ Other (12) ____

Q20 Do you believe that emphasizing one pillar of sustainability (environment, economic, or social) negatively impacts either of the other two?

- ◯ Yes (1)
- ◯ No (2)
- ◯ I do not know (3)

Q21 Why do you believe that emphasizing one pillar of sustainability (environment, economic, or social) negatively impacts either of the other two?

____

Q22 Why do you not believe that emphasizing one pillar of sustainability (environment, economic, or social) negatively impacts either of the other two?

____

Q23 Please choose one answer to fill in the blank in the following statement. Animal welfare __________ impacts environmental sustainability.

- ◯ Negatively (1)
- ◯ Does Not (2)
- ◯ Positively (3)

Q24 Please choose one answer to fill in the blank in the following statement. Animal welfare __________ impacts economic sustainability.

- ◯ Negatively (1)
- ◯ Does Not (2)
- ◯ Positively (3)

Q25 Please choose one answer to fill in the blank in the following statement. Animal welfare __________ impacts social sustainability.

- ○ Negatively (1)
- ○ Does Not (2)
- ○ Positively (3)

Q26 Do you have any additional comments to make regarding animal welfare, sustainability or about your program in general? Please type N/A if no additional comments.

____
